# Influence of Rescuers' Gender and Body Mass Index on Cardiopulmonary Resuscitation according to the American Heart Association 2010 Resuscitation Guidelines

**DOI:** 10.1155/2015/246398

**Published:** 2015-11-18

**Authors:** Ahmad Jaafar, Mohammad Abdulwahab, Eman Al-Hashemi

**Affiliations:** ^1^Department of Pediatrics, Mubarak Al-Kabeer Hospital, 46300 Jabriya, Kuwait; ^2^Faculty of Dentistry, Kuwait University, 46300 Jabriya, Kuwait; ^3^Faculty of Medicine, Kuwait University, 46300 Jabriya, Kuwait

## Abstract

*Background and Objectives*. The quality of cardiopulmonary resuscitation (CPR) is an important factor in determining its overall outcome. This study aims to test the association between rescuers' gender, Body Mass Index (BMI), and the accuracy of chest compressions (CC) as well as ventilation, according to American Heart Association (AHA) 2010 resuscitation guidelines.* Methods*. The study included 72 participants of both genders. All the participants received CPR training according to AHA 2010 resuscitation guidelines. One week later, an assessment of their CPR was carried out. Moreover, the weight and height of the participants were measured in order to calculate their BMI.* Results*. Our analysis showed no significant association between gender and the CC depth (*P* = 0.53) as well as between gender and ventilation (*P* = 0.42). Females were significantly faster than males in CC (*P* = 0.000). Regarding BMI, participants with a BMI less than the mean BMI of the study sample tended to perform CC with the correct depth (*P* = 0.045) and to finish CC faster than those with a BMI more than the mean (*P* = 0.000). On the other hand, no significant association was found between BMI and ventilation (*P* = 0.187).* Conclusion*. CPR can be influenced by factors such as gender and BMI, as such the individual rescuer and CPR training programs should take these into account in order to maximize victims' outcome.

## 1. Introduction and Objectives

In 1960, Kouwenhoven et al. reported that it is possible to sustain the life of a person with a cardiac arrest for as long as 30 minutes with a combination of external cardiac massage, also known as chest compressions, and mouth-to-mouth respiration [1]. These two components form what is known as cardiopulmonary resuscitation (CPR). CPR is considered as a critical part in the management of a cardiac arrest.

The quality of CPR is a major determinant of the survival from a cardiac arrest. In fact, it was revealed that inadequate CPR compromises patient's survival [2]. Additionally, it compromises the quality of life of those who survive, as it is linked with a poor neurological recovery [3]. Because of this, it is important to determine the factors that affect the overall quality of CPR.

The quality of CPR depends on the accuracy of its two main components, namely, chest compressions and ventilation. If the chest compressions were performed with an inaccurate depth, rate, or location or the ventilation delivered was inadequate to cause an appropriate chest rise, the overall quality of CPR will be negatively affected.

American Heart Association (AHA) 2010 resuscitation guidelines emphasize the importance of performing high-quality CPR and recommend that for CPR to be of a high quality the chest compression depth should be at least 2.5 inches, the chest compression rate should be at least 100/min, and the ventilation delivered should cause a noticeable chest rise.

This study aims to test the association between rescuers' gender, BMI, and the accuracy of chest compressions (CC), specifically the depth and rate, as well as ventilation (V), according to AHA 2010 resuscitation guidelines.

## 2. Methods

### 2.1. Study Design and Study Sample

This is a cross-sectional study in which 72 participants (30 males and 42 females) from Dasman's Clinical Skills Training Center meeting the inclusion criteria were randomly selected and included in the study. The inclusion criteria are age of 18 years or older, a healthcare provider or student, being healthy (no chronic disease like cardiac disease, respiratory disease, or physical disability), not being allergic to latex, and not receiving CPR training previously or at least for the past 2 years. The above inclusion criteria were set by the researchers in order to standardize the participants included in the study.

### 2.2. Data Collection

#### 2.2.1. Cardiopulmonary Resuscitation (CPR)

 The participants received CPR training by the same instructor according to AHA 2010 resuscitation guidelines. One week later, an assessment of their CPR was carried out. In the assessment, the participants were asked to perform 5 cycles of chest compressions and ventilation with a ratio of 30 : 2 on a manikin connected to Resusci Anne Basic and SkillGuide, a device that assesses the accuracy of chest compressions, in terms of their depth and location, as well as the accuracy of ventilation delivered.

In this device, whenever the rescuer performs the chest compression with the accurate depth (at least 2.5 inches) and location, a green-colored indicator appears. As the focus of the study was on the depth rather than the location of the chest compressions, an instructor had to make sure that the rescuer is compressing the chest in the right location.

As with chest compressions, a green-colored indicator will appear if the ventilation delivered was adequate to cause an appropriate chest rise. To make sure that there is no limitation to the airflow when delivering the ventilation, the manikin was positioned with a patent airway.

Each participant was assessed separately in a room with one manikin. The manikin was placed on a rigid backboard connected to the Resusci Anne Basic and SkillGuide device. Before each assessment, an instructor had to make sure that the device is functional.

Three certified BLS instructors were present during the assessment. The first instructor had to make sure that the rescuer is performing the chest compressions in the right location and that the manikin was positioned correctly to keep the airway patent; the second instructor had to inspect the device, gather data, and document them.

In order to measure the rate of chest compressions, the third instructor was given a stopwatch and was told to measure the total time taken to perform the chest compressions in the 5 CPR cycles excluding the time taken to deliver the ventilation. The chest compression rate was calculated by the following formula:

(1) $$\begin{array}{c} \text{Chest compression rate}=\frac{\text{Total number of chest compressions during the 5 CPR cycles }\left(\text{150}\right)}{\text{Total time taken to finish the 5 cycles of chest compressions in minutes}}\times 100. \end{array}$$

#### 2.2.2. Body Mass Index (BMI)

On the same day of the assessment, the weight and height of the participants were measured. BMI was calculated according to the following formula: BMI = (Weight in Kilograms/(Height in Meters × Height in Meters)).

### 2.3. Data Analysis

The Statistical Package for Social Sciences (SPSS Inc., Chicago, IL, USA, 2010) version 19 was used for data entry and analysis. *P* value ≤ 0.05 was used as the cut-off level for statistical significance. The nonparametric Chi-Square for independence test was used to explore the relationship between gender, BMI, and CPR, particularly the chest compression depth and rate as well as ventilation.

Regarding the chest compression depth, the five cycles of chest compressions were deemed effective when >80% of all compressions were of the correct depth. As for the chest compression rate, the five cycles of chest compressions were considered effective when the chest compression rate during the 5 CPR cycles was ≥100/min. On the other hand, the five cycles of ventilation were deemed effective when >80% of all ventilation delivered caused an appropriate chest rise. The body mass index figure of 26 was used as it was the mean BMI for the study sample. This will make comparison between two BMI groups more valid.

### 2.4. Ethical Considerations

An informed consent was obtained from each participant. It clearly stated that the participation in this study is completely optional and that there is no risk as a result of the participation in the study. In order to insure confidentiality, the names of participants or other identifying information were not obtained during the assessment. The research was approved by Dasman Diabetes Institute Review Board.

## 3. Results

Table 1 presents the sociodemographic characteristics of the study sample, namely, age and gender. The mean age of the study sample is 21 years with a standard deviation (SD) of 0.85. Out of the 72 participants, 30 (42%) are males, while 42 (58%) are females.

Table 2 presents the mean weight, height, and BMI of the study participants and their standard deviations.

Table 3 presents the number and percentage of the study participants with BMI more than and less than the mean BMI, which is 26.

Table 4 presents the association between gender and CPR, specifically the chest compression depth, chest compression rate, and ventilation. Regarding the chest compression depth, our analysis shows that 66.7% of males performed >80% of the chest compressions with the correct depth. For females, 76.2% performed >80% of the chest compressions with the correct depth. A Chi-Square test for independence indicates no significant association between gender and the percentage of chest compressions performed with the correct depth, *χ* ^2^ (1, *n* = 72) = 0.38, *P* = 0.53, and phi = 0.10. This means that the proportion of males who performed chest compressions with the correct depth is not significantly different from the proportion of females who performed chest compressions with the correct depth. Concerning the chest compression rate, the analysis shows that the chest compression rate during the 5 CPR cycles was 100/min or more in 40% of males. For females, 100% had that chest compression rate. A Chi-Square test for independence indicates significant association between gender and the chest compression rate: *χ* ^2^ (1, *n* = 72) = 30.476, *P* = 0.000, and phi = −0.68. From the previous result, one can conclude that the chest compression rate among males is statistically lower than the chest compression rate among females. As for ventilation, the results show that 40% of males delivered >80% of the ventilation correctly. For females, 52.4% delivered >80% of the ventilation correctly. A Chi-Square test for independence indicates no significant association between gender and the percentage of adequate ventilation delivered: *χ* ^2^ (1, *n* = 72) = 0.64, *P* = 0.42, and phi = 0.12. The result indicates that the proportion of males who delivered adequate ventilation is not significantly different from the proportion of females who delivered adequate ventilation.

Table 5 presents the association between BMI and CPR, specifically the chest compression depth, chest compression rate, and ventilation. In terms of the chest compression depth, the study shows that 81.8% of those with a BMI less than the mean BMI performed >80% of the chest compressions with the correct depth. For participants with a BMI more than the mean BMI, 57.1% performed >80% of the chest compressions with the correct depth. A Chi-Square test for independence indicates a significant association between BMI and the percentage of chest compressions with the correct depth: *χ* ^2^ (1, *n* = 72) = 4.03, *P* = 0.045, and phi = −0.26. The Chi-Square results show a value of 4.03, with an associated significance level of 0.045, hence indicating significant results. With regard to the chest compression rate, our analysis shows that the chest compression rate during the 5 CPR cycles is 100/min or more in 90.9% of those with a BMI less than the mean BMI. For participants with a BMI more than the mean BMI, 50% had that chest compression rate. A Chi-Square test for independence indicates that the chest compression rate among participants with a BMI more than the mean BMI is statistically different from the chest compression rate among participants with a BMI less than the mean BMI. As for ventilation, it was shown that 54.5% of those with a BMI less than the mean BMI delivered >80% of the ventilation correctly. For participants with a BMI more than the mean BMI, 35.7% delivered >80% of the ventilation correctly. A Chi-Square test for independence indicates no significant association between BMI and the percentage of adequate ventilation delivered: *χ* ^2^ (1, *n* = 72) = 1.74, *P* = 0.187, and phi = −0.18. The result indicates that the proportion of participants with BMI more than the mean BMI who delivered adequate ventilation is not significantly different from the proportion of those with BMI less than the mean BMI.

## 4. Discussion

Factors affecting CPR will ultimately affect its overall outcome, which is the victim's survival. In our study, we want to test the influence of both gender and BMI on CPR. CPR involves both chest compressions and ventilation and for the CPR to be of high quality both components need to be of high quality. According to AHA 2010 resuscitation guidelines, for CPR to be of high quality, the chest compression depth should be at least 2.5 inches, the chest compression rate should be at least 100/min, and the ventilation delivered should cause a noticeable chest rise.

### 4.1. Chest Compression Depth

A correct chest compression depth when performing CPR is one of the important aspects of high-quality CPR. Stiell et al. conclude that there is a strong association between survival outcomes and an increased compression depth [4].

The current study looks initially into the relationship between gender and the accuracy of CPR in terms of the chest compression depth. No significant association is found between gender and the percentage of chest compressions performed with the correct depth (*P* value = 0.5). Although the study results show 67% of males performed >80% of the chest compressions with the correct depth, while 76% of females performed similarly, such difference is found to be statistically not significant. Our study's results seem to contradict several previous studies that were conducted on the same issue. For example, a study that was conducted in the United States has shown that males were more effective than females in administering a sufficient compression depth [5]. Another study that was conducted in UK shows significant differences between males and females in achieving a sufficient compression depth, with males being decisively more effective in their performance throughout the whole CPR cycles [6].

Regarding the effect of BMI on the chest compression depth, it is found that participants with a BMI >26, which is the mean BMI of our sample, are less likely to achieve the correct depth when performing chest compressions (*P* value = 0.04). Our results show that 82% of those with a BMI <26 performed >80% of chest compressions with the correct depth, while only 57% of those with a BMI >26 performed at the same efficiency. This result contradicts the results of several previous studies. Reddy and his associates found that some positive correlations exist between the average adequate depth of chest compressions and BMI. Their results seem to be in line with Gianotto-Oliveira et al.'s study which claims that the depth of chest compressions is higher in the overweight people in comparison with normal weight and underweight people [7]. In addition, Sayee and McCluskey conclude that junior doctors in the UK with a BMI greater than 24, which is the mean BMI of their sample, are capable of more effective CPR, as judged by depth of chest compressions, when using a ratio of 30 : 2 [6]. Contrastively, our study shows the opposite results. This could be due to the tendency of having a slightly abnormal body positioning in overweight people (BMI > 26) while performing chest compressions. Another possible explanation could be an expected early fatigability in those with BMI >26, a factor that might have affected the accuracy of chest compressions in terms of their depth. Several studies suggest that rescuer's fatigue during the performance of 30 chest compressions may compromise the accuracy of CPR [8–10]. Such hypothetical explanations need to be investigated further in future studies.

While this study demonstrates the effects of gender and BMI on the chest compression depth, previous studies have investigated the impact of other factors on the chest compression depth including the position of the manikin [11] and the use of a backboard [12]. It is crucial to consider all these factors when performing chest compressions to maximize the victim's survival.

### 4.2. Chest Compression Rate

Sanders et al. suggest that an increased rate of chest compressions is associated with a better outcome. They compared the neurological outcome in pigs following an induced cardiac arrest and found better neurological recovery at higher rates of chest compressions [13]. On the other hand, Abella et al. have revealed that suboptimal compression rates are correlated with poor return of spontaneous circulation [14]. This is why AHA suggests that the rate of chest compressions should be at least 100/min. On the contrary, faster chest compressions do not necessarily mean better chest compressions. Although AHA does not mention in their latest guidelines an upper limit for the rate of chest compressions, 2010 European Resuscitation Council CPR Guidelines recommend an upper rate limit of 120 chest compressions/minute [15]. Additionally, Idris et al. [16] conclude that return of spontaneous circulation rates peaked at a compression rate of ≈125/min and then declined. This means that performing fast chest compressions can adversely affect CPR performance if the chest compression rate exceeds a specific limit [16].

Our study has looked into the relationship between the participant's gender and the rate of chest compressions. In this study, significant association is found between gender and the chest compression rate (*P* value = 0.000). Bearing in mind that the recommended chest compression rate by AHA in their latest guidelines is ≥100/min, the results of our study show that 40% of males had a chest compression rate of ≥100/min, while 100% of females had that chest compression rate. This indicates that females are significantly faster than males in performing chest compressions. Another study conducted by Russo et al. concludes that female rescuers are more rapid than male rescuers when performing chest compressions, with an average chest compression rate of 117/min in females [17]. However, Reddy et al. state that gender has no significant effect on chest compression rate [5].

A further investigated aspect in the current study is the association between BMI and the chest compression rate. The study reveals a statistically significant difference between participants with a BMI >26 and those with a BMI <26, with the former performing CPR compressions at a much slower rate. In this vein, Reddy and Gianotto-Oliveira found in their studies that there is no significant association between BMI and the chest compression rate.

One possible explanation of the study's finding could be the fact that females tend to have less BMI values than males. Further investigation needs to be done to adjust for such a confounding factor.

### 4.3. Ventilation

In the current study, an investigation of the relationship between the participant's gender and adequacy of CPR ventilation shows no significant association throughout the 5 CPR cycles. Such factor seems to be completely overlooked in the previous studies on the similar issue. As for the relationship between BMI and ventilation, the current study finds no statistically significant association between them. The aspect has also been overlooked in previous studies.

## 5. Conclusion

CPR can be influenced by factors such as gender and BMI. After a thorough analysis of the current study's results, a number of conclusions may be safely reached. Firstly, the chest compression rate is affected by both gender and BMI, as females as well as those with a BMI less than 26 tend to be faster in performing chest compressions. Secondly, BMI unlike gender tends to affect the depth of chest compressions leading to a more adequate performance amongst those with a BMI less than 26. Thirdly, ventilation was not affected by either gender or BMI. Further studies need to be conducted to further investigate the influence of such factors on CPR. Additionally, it is highly recommended that CPR training programs take these factors into account in order to maximize victims' outcome.

## Figures and Tables

**Table 1:** Sociodemographic characteristics of the 72 participants from Dasman's Clinical Skills Training Center meeting the inclusion criteria.

Sociodemographic characteristics	
Age in years, mean (SD)^*∗*^	21 (0.85)
Gender, *n* (%)	
Male	30 (42%)
Female	42 (58%)

^*∗*^Standard deviation.

**Table 2:** Weight, height, and body mass index (BMI) of the 72 participants from Dasman's Clinical Skills Training Center meeting the inclusion criteria.

Height in centimeters (cm), mean (SD)^*∗*^	164 (8.2)
Weight in kilograms (kg), mean (SD)^*∗*^	71 (23)
BMI, mean (SD)^*∗*^	26 (6.8)

^*∗*^Standard deviation.

**Table 3:** Body mass index (BMI) of the 72 participants from Dasman's Clinical Skills Training Center meeting the inclusion criteria.

Less than the mean BMI, *n* (%)	44 (61%)
More than the mean BMI, *n* (%)	28 (39%)

**Table 4:** Association between gender and CPR performance.

	Male	Female	*P* value
Percentage of participants achieving >80% of CC^1^ with the correct depth	67%	76%	0.5
Percentage of participants achieving >80% of V^2^ correctly	40%	52%	0.4
Percentage of participants with CC^1^ rate of ≥100/min	40%	100%	0.00

^1^Chest compressions.

^2^Ventilation.

**Table 5:** Association between body mass index (BMI) and CPR performance.

	BMI < 26	BMI > 26	*P* value
Percentage of participants achieving >80% of CC^1^ with the correct depth	82%	57%	0.04
Percentage of participants achieving >80% of V^2^ with the correct depth	54.5%	35.7%	0.1
Percentage of participants with CC^1^ rate of ≥100/min	91%	50%	0.00

^1^Chest compressions.

^2^Ventilations.
